# Preliminary validity evidence for a rapid fundamental movement skill assessment tool for primary education settings

**DOI:** 10.3389/fspor.2025.1441402

**Published:** 2025-04-14

**Authors:** Aaron Simpson, Brodie Ward, Michael Rosenberg, Ben Jackson, Jingdan Gou, Amanda Derbyshire, Ashleigh L. Thornton

**Affiliations:** ^1^ Department of Exercise and Sport Science, School of Human Sciences, The University of Western Australia, Perth, WA, Australia; ^2^ The Kids Research Institute Australia, Perth, WA, Australia; ^3^ Division of Paediatrics, School of Medicine, The University of Western Australia, Perth, WA, Australia; ^4^ Kids Rehab WA, Perth Children’s Hospital, Perth, WA, Australia

**Keywords:** motor competence, physical education, physical competence, motor development, elementary school

## Abstract

**Introduction:**

Assessment of motor competence is critical for planning and monitoring children's development. However, many assessment tools require time, training, and resources not available to most teachers. We aimed to evaluate the external aspect of construct validity of a rapid product-oriented fundamental movement skill assessment tool designed specifically for primary education settings.

**Methods:**

Fundamental movement skills of 73 children aged 4–8 were assessed using the KIDDO Challenge assessment tool and the Test of Gross Motor Development-2 (TGMD-2). We conducted correlational analyses between scores derived from the assessment tools.

**Results:**

We found significant associations (ranging from weak to moderate in nature; *r* range = −0.17 to 0.68) between scores of individual skills on the KIDDO Challenge and the TGMD-2. Age- and gender-standardised overall proficiency ratings between assessment tools exhibited a significant moderate, positive correlation (*r* = 0.52, *p* < .01).

**Conclusions:**

Our findings indicate that the KIDDO Challenge and TGMD-2 were significantly associated in their assessment of children's overall fundamental movement skill proficiency. These findings may assist teachers or school administration in selecting rapid fundamental movement skill assessment tools with evidence of validity for motor competence screening in primary education settings.

## 1 Introduction

The development of motor competence throughout early childhood has positive associations with life-long health behaviours and outcomes such as physical activity participation, physical fitness, weight status, and perceived physical competence (1–5). The relationship between motor competence and physical activity in childhood is posited to be synergistic and reciprocal, highlighting the salience of targeting motor competence and providing opportunities to engage in physical activity for child development (5). It is important, therefore, that children's motor competence can be assessed to track development and identify opportunities for early intervention (1, 6, 7). Motor competence broadly refers to one's ability to execute motor acts or “movements” (8). A popular approach to assessing gross motor competence is through the assessment of fundamental movement skills (FMS)—skills that provide the foundation for participation in a range of sports and activities, and that include running, jumping, catching, throwing, kicking, striking, and balance. Indeed, many consider FMS to be the “building blocks” for sports-specific skills (9).

A critical distinction between different FMS assessment tools is whether they utilise process- or product-oriented techniques (10). Process- and product-oriented assessments are posited to measure different aspects of motor competence (11). Process-oriented techniques allow for analysis of *how* skills are performed, and assess FMS in relation to motor patterns that contribute to proficient performance (12). A widely-used example of a process-oriented assessment tool employed by researchers is the Test of Gross Motor Development-2 (TGMD-2) (13). Process-oriented measures allow assessors to highlight technical and specific performance deficiencies to inform intervention strategies—however, they tend to be limited by administration burden and substantial training required for reliable administration (14). Assessment tools that utilise product-oriented techniques, on the other hand, provide assessors with insight into the *outcome* of FMS performances (e.g., distance or time). Subsequently, although product-oriented techniques are limited in their ability to highlight specific skill deficiencies, they tend to be more reliable, faster to administer, and require less training than process-oriented techniques (15). Given the large number of tools and techniques available, it is important that researchers and teachers consider assessment environments during selection of appropriate assessment tools (e.g., time constraints, administrator training) (1, 16). Additionally, certain tools may be more useful for specific purposes—process-oriented assessments, for example, were often developed with research or clinical purposes in mind [e.g., to identify children with deficiencies in motor competence; De Niet et al. (17)]. Many of these tools (that require more time and training to administer) have substantial validity evidence—consequently, researchers have called for more validation research for practical, easy-to-deliver product-oriented assessments (18).

Primary (i.e., elementary) education environments are central to the development and assessment of FMS. As a result of significant time and resource requirements, process-oriented assessments are often not feasible in the school environment, and teachers are typically unable to administer them accurately, reliably, and with high fidelity (12, 14, 19, 20). Researchers have suggested that more appropriate FMS assessment tools are required to ensure accurate and effective skill monitoring within educational settings, allowing for early intervention where required (21, 22). At present (and in lieu of rapid, cost-effective process-oriented assessments), product-oriented assessment techniques may offer feasible, rapid classification of motor competence in primary school settings. Because product-oriented tools assess FMS differently to process-oriented tools, results from product-oriented assessments may improve teachers' capacity to screen their classes and better identify children with motor deficiencies. In turn, these children can then undergo a more comprehensive process-oriented assessment to identify and address specific challenges with their movement patterns (i.e., *how* their FMS are performed). There is mixed evidence regarding the validity of product-oriented assessments in schools—the most frequently researched tool is the Movement Assessment Battery for Children [for a review of validity and reliability studies for FMS tools, see Eddy et al. (23)]. This tool—and other similar product-oriented assessments—also often require intensive time and resource (e.g., training) commitment, limiting their feasibility in large-scale school settings (6, 24). Researchers are, in recent years, dedicating more attention to the feasibility of FMS assessment tools in non-clinical (e.g., school) settings; however, it remains that many of the most popular tools are associated with challenges related to feasibility (25). Some product-oriented tools, however, have shown promise for being quick and easy to use alongside evidence of validity [e.g., the Athletic Skills Track (26, 27)]. The KIDDO Challenge, a product-oriented assessment tool, was developed to address the need for rapid FMS assessment in schools that can be implemented with little training or equipment (see below for a description).

An important feature of providing validity evidence for assessment tools is to demonstrate associations with other measures of the same (or similar) construct. This is often referred to as the external aspect of construct validity [more specifically, convergent correlations, see Messick (28)]. The purpose of the present study was to provide preliminary evidence for the validity of data obtained from a rapid product-oriented FMS assessment tool (the KIDDO Challenge) for primary school-aged children. In assessing the external aspect of construct validity, we can provide evidence on whether FMS proficiency scores derived from KIDDO Challenge are associated with scores from a more established and widely-used FMS assessment tool (the TGMD-2). Despite recent evidence that product- and process-oriented tools assess different aspects of motor competence (11), the inclusion of both “types” of assessment tool in this study allows us to consider the suitability of rapid product-oriented assessments in school settings (compared with process-oriented tools with longer and more complex administration requirements). By extension, we aim to provide preliminary evidence to support the use of rapid product-oriented FMS assessment tools as a solution to the challenges faced in primary school education settings.

## 2 Methods

### 2.1 Participants

One hundred and twenty-one children between the ages of 4 and 8 from two primary schools in Perth, Western Australia, participated in the study. Forty-eight participants were excluded from analyses due to not completing both assessments, or incorrect completion of the TGMD-2 assessment, resulting in a final sample of 73 children. The two participating schools were in the public (i.e., Government) school system in the metropolitan area of Perth; one was situated in a high socioeconomic area, and the other in a low socioeconomic area. Children with a physical disability or health condition that prevented them participating in the standardised FMS assessments were excluded from participating in the study. We obtained written informed consent from legal guardians and verbal assent from participants prior to data collection. This study received approval by the Human Research Ethics Board of the lead author's institution and the Western Australian Government Department of Education.

### 2.2 Instruments

The TGMD-2 is a widely used process-oriented FMS assessment tool. The TGMD-2 includes 12 FMS across two subtests (locomotor and object control; see Table 1 for a list of all skills assessed in the present study). Each skill is assessed based on the presence or absence of between three and five criteria pertaining to proficient performance of the skill—for example, for the run assessment, criteria include (i) moving arms in opposition to legs with elbows bent, (ii) a period where both feet are off the ground, (iii) narrow foot placement landing on heel or toe, and (iv) non-support leg bent to approximately 90 degrees (13).

**Table 1:** TGMD-2 refers to the test of gross motor development-2; GSGA refers to the get skilled, get active assessment tool. Only the balance assessment from the GSGA was performed in this study. It should be noted, however, that the GSGA assesses other FMS when completed in full.

Skill classification	KIDDO challenge	TGMD-2	GSGA
Locomotor			
Run	✓	✓	
Jump	✓	✓	
Gallop		✓	
Hop		✓	
Leap		✓	
Slide		✓	
Object control			
Bounce & catch	✓		
Strike		✓	
Dribble		✓	
Kick	✓	✓	
Catch		✓	
Overhand throw		✓	
Underhand roll		✓	
Stability			
Balance	✓		✓

The KIDDO Challenge is a rapid product-oriented assessment tool developed by researchers within the KIDDO physical literacy research group (see KIDDO.edu.au). The KIDDO Challenge is used to assess outcome scores on five FMS tasks—two locomotor skills (run, jump), two object control skills (kick, “bounce and catch”), and a stability skill (balance). Running (a 50 metre sprint) and balancing (single-leg balance task for a maximum of 40 second per leg) are assessed via time. Jumping (standing broad jump) and kicking (soccer kick) are assessed via distance. Bouncing and catching is assessed via a two-hand bounce and catch task with a basketball—with the assessor counting the number of *catches* in a 20 second period. Some aspects of the KIDDO Challenge were adapted from an FMS assessment tool with preliminary validity and reliability evidence (Stay in Step assessment tool) (29). The KIDDO Challenge was designed to allow teachers to assess FMS within the time and resource constraints of educational settings, allowing proficiency classification and the recognition of children who may require more comprehensive assessment.

As the TGMD-2 does not include a stability task, the static balance task from the Get Skilled, Get Active (GSGA) (30) assessment tool was utilised as an established process-oriented stability assessment. The static balance task within the GSGA includes five proficiency criteria for assessment. GSGA administration typically involves five trials per skill—however, in the present study, two trials were collected and scored to remain consistent with the TGMD-2 and KIDDO Challenge.

### 2.3 Assessment administration

Administrators of the assessments were recruited via email communication from the lead author. All administrators were involved as coaches within the KIDDO physical literacy program and either held a degree in Exercise and Health Science or were in their final year of study. All had experience in FMS instruction and assessment for children of all abilities, across multiple motor development programs. Administrators were trained by an expert in the field of FMS research administration who has experience with both the TGMD-2 and the KIDDO Challenge. Administrators were trained in a two hour training session, and were familiarised with the protocols for administering each assessment tool (including set-up, instruction, and demonstration). Administrators were familiarised with the scoring procedures of the KIDDO Challenge—however, because TGMD-2 and GSGA performances were to be filmed for *post-hoc* assessment, administrators were not trained in the scoring procedures for these tools. Once assessors had been familiarised with the tools, each administrator conducted three practice assessments to ensure they understood the process.

Assessments were administered on the school oval (grass surface), or concrete area adjacent to the school oval (for the KIDDO Challenge bounce and catch/TGMD-2 dribble task), of each school. Each participant completed the respective assessments one week apart—we randomised the order of assessment for each participant to ensure that each tool was administered at both timepoints across the sample. KIDDO Challenge scores were recorded during administration within the Qualtrics (Qualtrics, Provo, UT) survey platform on a tablet smart-device, and TGMD-2 (and GSGA Balance) assessments were filmed using video cameras for *post-hoc* assessment. Participant's height and weight were also collected during administration of the KIDDO Challenge. For each skill assessment (except for the KIDDO Challenge run assessment), the administrator demonstrated the skill, then allowed the participant to complete a practice trial, followed by two formal trials. If it was clear the child had not understood the instruction, a second demonstration was performed. For the KIDDO Challenge run task, a single formal trial was administered (with no practice trial, to minimise risk of fatigue impacting results). The KIDDO Challenge balance task has a 40 second ceiling (i.e., assessment stops at 40 second) on each leg—if a child maintained their balance for 40 second on the first trial for that leg, a second trial was not administered. Therefore, the minimum number of trials for the KIDDO Challenge balance assessment was two (one on each leg), and the maximum was four (two on each leg). More specific guidance on how the KIDDO Challenge is administered is available in Supplementary Material 1.

TGMD-2 performances were assessed *post-hoc* by three assessors. All assessors had also taken part in the administration component of the study, and also completed additional TGMD-2 assessment training. The training included familiarisation with the TGMD-2 criteria for each skill and their interpretation, and three guided TGMD-2 assessments with an FMS expert. The three assessors then independently completed assessments until agreement exceeded 90% for each proficiency criterion across three consecutive assessments. Performances were then divided between the three assessors for independent assessment.

### 2.4 Data analysis

We conducted our analyses in five stages. First, we assessed the structural aspect of construct validity (28). According to Messick's (28) conceptualisation of construct validity, the structural aspect of validity refers to the fidelity of the scoring structure—we conducted a principal components analysis to assess this prior to examining the external aspect of construct validity. Our principal components analysis was guided by recommendations outlined by Comrey and Lee's (31); loading coefficients above .5 were considered as acceptable.

Second, we used Spearman rank order correlations to assess relationships between raw scores for each skill in the KIDDO Challenge and TGMD-2 (and GSGA for balance). TGMD-2 and GSGA raw scores were represented by total number of proficiency criteria performed for each skill across 2 trials. KIDDO Challenge raw scores were represented by the best score of the two trials for each skill (except for the run, where one trial was performed, and balance, calculated as the sum of the best balance score on each leg).

Third, we standardised scores for both assessments by age and gender to allow analysis of the strength of association between overall proficiency in both assessments. We converted TGMD-2 raw scores to standard scores for the locomotor and object control subsets, and gross motor quotients following the procedures outlined in the test manual (13).

Fourth, we standardised KIDDO Challenge scores by age and gender, and into five cut-points based on normative data for each task. We established normative cut-points using data from 6,599 KIDDO Challenge assessments [3,464 girls, 3,135 boys, mean age = 5.5 (SD = 1.28) years] of primary school-age children collected from 2017 to 2020. Each child's overall KIDDO Challenge proficiency (used as a comparison with TGMD-2 standard scores and GMQ) was determined by summing the standardised cut-point scores (scored 1–5) for all five KIDDO Challenge tasks, providing an overall proficiency score out of 25. Proficiency for KIDDO Challenge locomotor performance was calculated as the summed standard scores for the jump and run tasks. Object control proficiency for the KIDDO Challenge was calculated as the summed standard scores for the kick and bounce & catch tasks.

Finally, we calculated strength of association between age- and gender-standardised proficiency across both assessments using Pearson product-moment correlations.

## 3 Results

Of the 73 children in the study, 33 were female and 40 were male. The mean age across the sample was 5.97 (SD = 0.85). Across the sample, three participants were 4 years old (*n*_male_ = 2), 17 were 5 years old (*n*_male_ = 10), 33 were 6 years old (*n*_male_ = 16), 19 were 7 years old (*n*_male_ = 11), and one was 8 years old (*n*_male_ = 1).

Findings from our principal components analysis revealed that all five KIDDO Challenge assessment items—run (loading coefficient = −.513), jump (.793), kick (.723), balance (.603), and bounce and catch (.792)—loaded onto one principal component, which explained 48% of variance. This provides preliminary support for the structural aspect of construct validity of FMS assessments measured using the KIDDO Challenge.

For context on the FMS performance of the sample, descriptive statistics of raw scores for individual skills are presented in Table 2. Spearman rank order correlations between raw scores for individual skills indicated weak (e.g., run, *r* = −0.17) to moderate-strong (e.g., bounce and catch, *r* = 0.68) associations between the TGMD-2 and KIDDO Challenge. In Table 3, we present the strength of association for all individual skills. For the running assessments, a negative correlation is observed due to a lower sprint time representing higher proficiency in the KIDDO Challenge, whereas TGMD-2 scores increase with respective proficiency. All associations (except for running) were statistically significant, although weak.

**Table 2:** Descriptive statistics of skill scores within each assessment.

Assessment tool	Mean score (SD)						
	Jump	Run	Kick	Balance	Bounce & catch	Dribble	Catch
KIDDO challenge	108.6 (23.35)	11.55 (1.58)	15.6 (6.56)	36.96 (25.01)	14.58 (7.08)		
TGMD-2	4.19 (2.08)	7.42 (0.90)	6.33 (1.52)			4.75 (2.61)	4.12 (1.50)
GSGA				6.59 (3.52)			

Units for the KIDDO challenge scores: Jump, centimetres; run, seconds; kick, metres; balance, seconds (to a maximum of 80); bounce and catch, number of catches.

**Table 3:** Spearman rank order associations of individual skills across product- and process-oriented assessments.

	TGMD-2					GSGA
KIDDO Challenge	Jump	Run	Kick	Dribble	Catch	Balance
Jump	*r =* 0.23* (*p* = .046)					
Run		*r =* 0.17 (*p =* .158)				
Kick			*r =* 0.34** (*p =* .003)			
Bounce & Catch				*r =* 0.68** (*p <* .001)	*r =* 0.38** (*p =* .001)	
Balance						*r =* 0.47** (*p <* .001)

*Denotes *p* < 0.05.

**Denotes *p* < 0.01.

Both the KIDDO Challenge and TGMD-2 assessment tools were similar in their classifications of overall proficiency, with significant moderate associations. Standardised for age and gender, the strength of association was highest between overall proficiency in the KIDDO Challenge and TGMD-2 Gross Motor Quotient (*r* = 0.52, *p* < .01). The association between overall proficiency in the KIDDO Challenge and TGMD-2 summed standard scores was similar (*r* = 0.50, *p* < .01). The association between scores on locomotor skills (*r* = 0.37, *p* < .01) and object control skills (*r* = 0.42, *p* < .01) assessed using the KIDDO Challenge and TGMD-2 was low-moderate.

## 4 Discussion

FMS assessment tools are critical for children's FMS development—however, there are few FMS assessment tools with evidence of validity that are feasible for rapid implementation in primary school settings. In the present study, we aimed to provide evidence for the external aspect of construct validity of a rapid FMS assessment (the KIDDO Challenge) designed to be feasible within the constraints of primary schools.

Our analyses revealed significant moderate correlations of age- and gender-standardised proficiency outcomes, indicating that the KIDDO Challenge FMS assessment generally scores children's FMS proficiency (as a whole) in alignment with the TGMD-2. At an individual skill level, correlations were in most cases weaker. The lack of strength in observed correlations may be a result of the KIDDO Challenge test itself, differences in *what* product- and process-oriented tools measure, or study-level issues (e.g., sample size). Although the TGMD-2 is not necessarily a gold-standard measure [nor is any FMS assessment (32)], these low correlations between skills assessed by the TGMD-2 (and GSGA) and KIDDO Challenge require further testing. Scores derived from the jump and run assessments in particular were weak. In concordance with this finding, Logan et al. (15) reported that locomotor skills (jumping in particular) may have weaker associations across product- and process-orientated assessments. Logan et al. (15) attributed weaker associations to potential ceiling effects—a limitation of process-orientated assessments. A ceiling effect was observed in the present study—specifically, in the run assessment of the TGMD-2, where the majority of the sample (*n* = 49) achieved the maximum score and the average score was 7.42 (out of a possible 8). However, there are also potential issues worth exploring for the run assessment of the KIDDO Challenge—for example, it could be argued that time to complete a 50 metre run is not a suitable measurement of FMS proficiency (and it may be confounded by other developmental factors). Regardless, for the other skills and for overall proficiency, where the significant associations observed in this study support the validity of FMS results from the KIDDO Challenge, and by extension, the use of this tool to assess FMS in schools.

We elected to evaluate FMS assessments measured using the KIDDO Challenge (a product-oriented assessment tool) against the TGMD-2 (a process-oriented assessment tool) to determine whether it could be used as a rapid screening tool in environments where it is not feasible to implement in-depth process-oriented FMS assessment for every child. Some researchers have posited that product- and process-oriented assessments represent different measurements of an equivalent concept (i.e., motor competence), instead of measurement of equivalent performance aspects (11, 15, 33). In support of this argument, researchers have suggested that changes in movement patterns captured by a discrete number of proficiency criteria may not reflect changes in movement outcomes captured by a product-oriented assessment, such as the KIDDO Challenge (11, 15). Additionally, increases in product-oriented performance as a result of biomechanical variables (e.g., timing, angular velocity) and neuromuscular mechanisms may not be captured within the binary “presence” or “absence” of proficiency criteria (34, 35). Importantly, FMS assessment is most commonly undertaken in a primary school environment, where there is a lack of time and confidence to administer assessments such as the TGMD-2. These barriers may result in teachers not undertaking or delaying FMS screening (36)—evidence for the use of rapid product-oriented assessments may encourage teachers to use tools like the KIDDO Challenge to screen children's FMS and identify students who may require additional support. The KIDDO Challenge was developed to provide teachers with a rapid FMS assessment option to increase uptake of FMS assessment in the time-poor primary school environment, where FMS assessment may not have previously occurred. In line with the notion that any assessment of FMS is better than *no* FMS assessment, we encourage the use of rapid FMS assessment tools like the KIDDO Challenge—particularly in school settings where significant resource barriers exist. Alongside this recommendation, we suggest further and more detailed examination of validity and reliability of these tools. Additionally, for the KIDDO Challenge specifically, more work is required on establishing standardised norms to enable the classification of children based on their overall score [see (37) for an example of this for a product-oriented tool]. Expanding the evidence base for rapid product-oriented FMS tools may have important implications for the uptake of FMS assessment in schools.

In recent work, Palmer et al. (11) identified that correlations between product- and process-orientated assessments before and after a 13-week FMS intervention were lower at post-test than pre-test. Their findings support the notion that throughout early years of FMS development, movement patterns and resultant outcomes develop independently to some extent. Targeted interventions to improve movement patterns from those previously established prompts disruption to the dynamic system (e.g., relative timing, segmental interactions), and observed changes to movement outcomes across the same period will depend on whether the intervention period was sufficient for the co-ordination system to re-organise and optimise (26, 38). It is important to note that either assessment approach is not necessarily favourable over the other—instead, assessments are considered to measure disparate aspects of performance (a notion further propelled by the weak correlations between individual FMS scores obtained from product-oriented and process-oriented tools in this study). Subsequently, test administrators should consider what they want to derive from FMS assessment when selecting an appropriate tool for their application. Or, whether multiple different tests are required—which would align with current evidence (39), but comes with logistical challenges and barriers (36). There is also some promising recent evidence for hybrid (i.e., product- and process-oriented) assessment tools (40, 41), and we recommend further exploration of rapid FMS assessments that utilise both approaches.

In the present study, we contribute to the literature pertaining to product- and process-oriented FMS assessments by examining associations between the approaches (for the purpose of providing construct validity evidence for FMS assessments from the KIDDO Challenge). It is also a strength of this work that the FMS assessment used addresses some of the practical limitations faced in the school environment while also assessing all domains of FMS (locomotor, object control, and stability). However, it is important to acknowledge the limitations of this study (and relevant future directions). First, although the sample was appropriate for the purpose of this study, it was relatively small and only included representation from two schools. Our sample may have limited the generalisability of our findings or the strength of observed correlations. And, our small sample size limited our ability to conduct other analyses that may be of value (e.g., associations between age groups and gender to assess the validity evidence for this tool across age groups, or the establishment of norms to classify motor proficiency). Given that preliminary construct validity evidence for the KIDDO Challenge exists, future research should be conducted with a larger and broader sample to provide additional empirical support for the validity of FMS scores obtained from the KIDDO Challenge. Additionally, the cross-sectional nature of the study should be noted, and associations between the TGMD-2 and KIDDO Challenge presented may not be reflective of the ability of the KIDDO Challenge to discriminate changes in performance over time. Finally, reliability of the KIDDO Challenge was not analysed. In future work establishing the KIDDO Challenge as a feasible and reliable assessment tool, researchers should consider exploring test-retest and inter-rater reliability, as well as the assessments’ sensitivity to proficiency changes over time. And, further examination of other aspects of validity, including analysis “against” other product-oriented tools or more sophisticated and explicit in-depth analyses of factor structure (especially given the loading coefficient of the run score in this study) is warranted.

Primary education environments remain a central site of FMS development across the early years, with FMS assessment providing teachers with the means to plan and monitor their programs. Assessment developers need to acknowledge the considerable time, training, and resource constraints within the school and early childhood environments. In many cases, these factors limit the feasibility of process-oriented assessments favoured in research settings (14). To overcome the constraints of the school environment, it is critical to establish validated FMS assessments that provide feasible and effective opportunities to monitor children's development with specific consideration of administration environments. We provide evidence for the external (and structural) aspect of construct validity in regard to assessing the movement proficiency of children aged 4–8 using the KIDDO Challenge assessment tool. In the present study we highlight the potential for utilising rapid product-oriented assessment tools in widespread screening of FMS in schools, which presents opportunities for efficient FMS monitoring and assessment, enabling earlier detection of, and intervention for, children at risk of developmental delay.

## Data Availability

The raw data supporting the conclusions of this article will be made available by the authors, without undue reservation.
