# Supplementary material for: Preliminary validity evidence for a rapid fundamental movement skill assessment tool for primary education settings
Source: Front Sports Act Living. 2025 Apr 14;7:1441402. doi: 10.3389/fspor.2025.1441402 (PMC12034640; doi:10.3389/fspor.2025.1441402)

Supplementary Material 1: KIDDO Challenge Guide

Five skills are assessed:

- Static balance
- Bounce and catch
- Standing broad jump
- Run
- Kick

These skills can be completed in any order.

Equipment List:

- 1x Basketball or equivalent (Size 5)
- 2x Soccer ball (size 4)
- 1x Stopwatch
- 20x Dome Markers/Cones
- 1x flat marker
- 1x 50m measuring tape

1. Balance

Task Description: The task requires the child to balance on one leg for as long as possible, with their hands on their hips

Equipment: Stopwatch

Environment: Flat, hard surface (e.g. concrete)

Demonstration:

- Upright posture
- Hands remain on hips
- Non-support foot held away from support leg

Starting Position: Hands are placed on hips and weight is on the preferred foot ready. Start the stopwatch when they are ready to lift their foot off the ground.

Test: Two trials may be given for each leg. The second trial is given only if the child scores less than 40 seconds on the first trial.

Timing is stopped if:

- The non-support leg touches the floor in order to recover balance
- The child moves about (hops or slides) on the support leg in order to retain balance
- The non-support leg rests on the support leg for added hip stability
- One or both hands are removed from the hip in order to regain balance or prevent failing; or
- The child balances for 40 seconds

Scoring:
Record the times for all balance attempts. The score is the best time for each leg added together.

2. Bounce and Catch

Task Description: The child is required to bounce and catch the ball with both hands as many times as possible in 20 seconds.

Equipment: Basketball (Size 5), stopwatch

Environment: Flat, hard surface (e.g. concrete)

Demonstration:

- Ball is caught each time and bounced—not just allowed to drop
- No pat bounce action (i.e., ‘slapping’ the ball)
- Ball is caught at approximately waist level
- Upright posture

Starting Position: Stand in an upright position holding the ball with both hands at waist level.

Test: One timed trial of 20 seconds

Scoring:
Record the number of catches in 20 seconds

Additional Rules:

- A deflected ball should be retrieved and bouncing continued until the time is up, no additional time is given to collect the ball (misdirection of the ball will result in the ball bouncing on the feet or out of reach, mistiming the catch may result in fumbling or dropping the ball).

3. Jump

Task description: The child is required to perform a two-footed jump as far as they can from a set start position.

Equipment needed: Tape measure, flat marker (not essential – a line indicating the start position will suffice).

Environment: Grass

Demonstration:

- Crouch during preparation
- Arms swinging back during prep, fully extending in air, and outstretched forward during landing
- Taking off and landing on two feet simultaneously and remaining still upon landing

Starting Position: Child stands on the marker behind start line

Test: Two trials

Scoring:
Record the distance (in cm) from the marker at the start position to **the heel of the back foot** for both trials. The furthest distance is used for the final score.

Additional Rules:

- If the participant falls over or moves immediately after landing, the trial is invalid and should be repeated.
- If the child takes off or lands on one foot, the trial is invalid and should be repeated.
- If the child cannot perform a valid jump after a secondary demonstration and additional attempt, they should receive a score of **0.**

4. Run

Task Description: The child is required to run as fast as possible for 50 metres between the designated markers.

Equipment needed: Cones or markers to mark start and finish lines, stopwatch, tape measure

Environment: Grass

Set-up: Place two markers 1.5 metres apart to make a starting line, measure 50 metres from the start line and place a marker to mark the finish line. Place markers every 10 metres to provide a guide for the child to run next to.

Demonstration:
- Alternating movement of arms and legs
- High knee lift (forward stroke), and 90 degree knee bend (backward stroke) of non-support leg
- Large stride where both feet are off the ground
- Upright posture

Starting Position: Stand in a ready position with one foot placed behind the starting line.

Test: One trial

Scoring:
- The time taken to complete the distance to the finish line is recorded (to two decimal places).

5. Kick

Task Description: The child is required to kick a soccer ball as far as they can.

Equipment needed: 2x Size 4 soccer balls, 5x markers, tape measure

Environment: Grass

Set-up: Create a box with four markers, 3 metres-long by 2 metres-wide. Measure 30 metres from the box in a straight line in the direction you want children to kick the ball and place a marker there (to indicate direction). Leave tape measure extended out to the marker. If you don't have a long tape measure, place markers every 5 metres up to 30 metres, and you can then measure kick distance with a smaller tape measure from the nearest cone to where the ball ends up.

Demonstration:
- The child can start anywhere within the starting box, but the ball is to be placed on the 'front' line of the box (i.e., the side where the target marker and tape measure are set up)
- Rapid and continuous run-up
- Elongated stride before kick
- Support leg placed next to ball
- Kick as hard as possible

Starting Position: Stand in an upright position on mark on the ground, within the starting box.

Test: Two trials

Scoring:
- The furthest kick out of two trials measured to the back (i.e., closest side to the starting position) of the ball to the closest centimetre. The measurement should be taken from the ball's final resting position.
- If the ball is kicked at an angle, the measurement is taken from the tape in line with the ball's final position as per the diagram below.

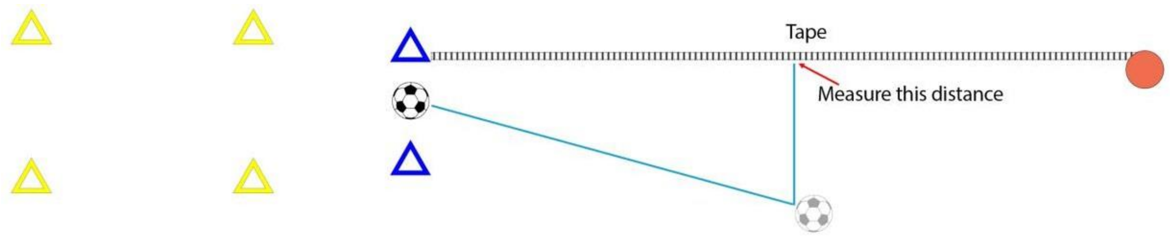

Supplement: Supplementary file 1 [file Datasheet1.pdf]
